# Increased Cardiac Myocyte PDE5 Levels in Human and Murine Pressure Overload Hypertrophy Contribute to Adverse LV Remodeling

**DOI:** 10.1371/journal.pone.0058841

**Published:** 2013-03-18

**Authors:** Sara Vandenwijngaert, Peter Pokreisz, Hadewich Hermans, Hilde Gillijns, Marijke Pellens, Noortje A. M. Bax, Giulia Coppiello, Wouter Oosterlinck, Agnes Balogh, Zoltan Papp, Carlijn V. C. Bouten, Jozef Bartunek, Jan D'hooge, Aernout Luttun, Erik Verbeken, Marie Christine Herregods, Paul Herijgers, Kenneth D. Bloch, Stefan Janssens

**Affiliations:** 1 Department of Cardiovascular Sciences, KU Leuven, Leuven, Belgium; 2 Department of Biomedical Engineering, Eindhoven University of Technology, Eindhoven, The Netherlands; 3 Division of Clinical Physiology, Institute of Cardiology, Research Center for Molecular Medicine, University of Debrecen Medical and Health Science Center, Debrecen, Hungary; 4 Cardiovascular Center, OLV Hospital, Aalst, Belgium; 5 Department of Imaging and Pathology, KU Leuven, Leuven, Belgium; 6 Anesthesia Center for Critical Care Research, Cardiovascular Research Center, Massachusetts General Hospital and Harvard Medical School, Boston, Massachusetts, United States of America; Leiden University Medical Center, The Netherlands

## Abstract

**Background:**

The intracellular second messenger cGMP protects the heart under pathological conditions. We examined expression of phosphodiesterase 5 (PDE5), an enzyme that hydrolyzes cGMP, in human and mouse hearts subjected to sustained left ventricular (LV) pressure overload. We also determined the role of cardiac myocyte-specific PDE5 expression in adverse LV remodeling in mice after transverse aortic constriction (TAC).

**Methodology/Principal Findings:**

In patients with severe aortic stenosis (AS) undergoing valve replacement, we detected greater myocardial PDE5 expression than in control hearts. We observed robust expression in scattered cardiac myocytes of those AS patients with higher LV filling pressures and BNP serum levels. Following TAC, we detected similar, focal PDE5 expression in cardiac myocytes of C57BL/6NTac mice exhibiting the most pronounced LV remodeling. To examine the effect of cell-specific PDE5 expression, we subjected transgenic mice with cardiac myocyte-specific PDE5 overexpression (PDE5-TG) to TAC. LV hypertrophy and fibrosis were similar as in WT, but PDE5-TG had increased cardiac dimensions, and decreased dP/dt_max_ and dP/dt_min_ with prolonged tau (*P*<0.05 for all). Greater cardiac dysfunction in PDE5-TG was associated with reduced myocardial cGMP and SERCA2 levels, and higher passive force in cardiac myocytes *in vitro*.

**Conclusions/Significance:**

Myocardial PDE5 expression is increased in the hearts of humans and mice with chronic pressure overload. Increased cardiac myocyte-specific PDE5 expression is a molecular hallmark in hypertrophic hearts with contractile failure, and represents an important therapeutic target.

## Introduction

Sustained left ventricular (LV) pressure overload, as in aortic stenosis (AS), causes pathological hypertrophy, reactivation of a fetal gene program, and maladaptive molecular alterations that initiate transition to heart failure [1]. Accumulating evidence points to a cardioprotective role of the ubiquitous intracellular second messenger cyclic guanosine 3', 5'-monophosphate (cGMP) in this pathological response [2]. In the cardiovascular system, cGMP is generated by guanylate cyclase isoforms in response to natriuretic peptides and nitric oxide, and its biological actions are predominantly mediated by cGMP-dependent protein kinases (PKG) and phosphodiesterases (PDE).

Of the 11-member PDE superfamily, PDE 1, 2, 3, 5, and 9 hydrolyze cGMP in the heart to relatively inactive GMP [3]. PDE 1, 2, and 3 exert dual-substrate specificity for cGMP and cyclic adenosine 3', 5'-monophosphate (cAMP), while PDE5 and PDE9 are cGMP-specific. The most widely studied cGMP-esterase is PDE5, which is prominently expressed in vascular smooth muscle cells (VSMC), and is therapeutically targeted in the treatment of erectile dysfunction and pulmonary hypertension [4], [5]. Under normal physiological conditions, PDE5 expression in the heart is low, but, in human right ventricular hypertrophy and advanced LV failure, PDE5 is increased in cardiac myocytes, suggesting that the enzyme has a role in the adaptation to increased stress [6], [7], [8]. Treatment with sildenafil, a selective PDE5 inhibitor, attenuated LV remodeling in mice subjected to TAC, and was recently shown to confer benefit in patients with stable systolic heart failure and in heart failure patients with preserved ejection fraction and pulmonary hypertension [9], [10], [11]. However, it is possible that some of sildenafil's cardiac effects are attributable to inhibition of PDE1 [12]. In view of the wide expression of PDE1 in the cardiovascular system, including in cardiac myocytes, possible cross-reactivity is relevant [13]. Moreover, beneficial effects of PDE5 inhibitors on cardiac remodeling could involve targeting of other cardiac cell types including fibroblasts, smooth muscle cells, and endothelial cells, or modulation of LV afterload via vasodilatation of systemic resistance vessels.

In the present study, we investigated spatial PDE5 expression patterns in myocardial tissue of patients with severe aortic stenosis undergoing aortic valve replacement (AVR). We observed marked PDE5 expression in scattered cardiac myocytes of patients with increased LV filling pressures. We report a strikingly similar PDE5 expression in cardiac myocytes of mice with the most pronounced cardiac remodeling in response to transverse aortic constriction (TAC)-induced chronic pressure overload. Finally, by using a transgenic approach, we show that cardiac myocyte-restricted increased PDE5 expression during sustained pressure overload contributes to contractile dysfunction and adverse LV remodeling.

## Materials and Methods

### Ethics Statement

The patient study was performed in accordance with the Declaration of Helsinki, and approved by the Medical Ethics Committee of University Hospital Gasthuisberg (Leuven, Belgium) or the Ethics Committee of OLV Hospital (Aalst, Belgium). All patients provided written informed consent.

Animal research was conducted according to the 2010/63/EU Directive, and approved by the Ethical Committee for Laboratory Experimentation of KU Leuven (Leuven, Belgium - permit number: P078/2007).

### Aortic Stenosis Patients

Cardiac tissue samples (n = 20) were obtained from the LV outflow tract of patients with isolated severe aortic stenosis and chronic pressure overload during AVR surgery at University Hospital Gasthuisberg (Leuven, Belgium) or OLV Hospital (Aalst, Belgium). PDE5 expression was analyzed using immunohistochemistry (see File S1 for details).

### Experimental Animals and TAC Procedure

Mice with cardiac myocyte-specific overexpression of PDE5 (PDE5-TG) were backcrossed for 6 generations onto a C57BL/6NTac (Taconic Inc) background [8]. Wild-type littermates served as controls. As reported previously, PDE5 protein expression was 9-fold higher and sildenafil-inhibitable cGMP hydrolysis 10-fold greater in PDE5-TG than in WT LV tissue (*P*<0.05 for both) [8]. Myocardial cyclic nucleotide concentrations and expression of endogenous PDE isoforms at baseline did not differ between PDE5-TG and WT [8].

Acute and chronic pressure overload was induced by TAC (using a 27 gauge needle) in PDE5-TG and WT male age- and body weight-matched mice. Anesthesia was induced with sodium pentobarbital (Ceva Santé Animale, 40–70 mg/kg IP), of which the depth was controlled by monitoring muscle tone (toe pinch) during the entire procedure. An analgesic (buprenorphine, Schering-Plough, 0.1 mg/kg SC) was administered during the first two days after surgery (for details see File S1).

### Cardiac Imaging and Hemodynamic Measurements

Ten weeks after TAC, transthoracic echocardiography (TTE) was used to measure end-diastolic and end-systolic dimensions and volumes (volume = π*LVID^3^/6, with LVID indicating LV internal diameter at the level of the papillary muscles), fractional shortening, and ejection fraction. Invasive pressure measurements were used to determine heart rate, transaortic pressure gradient, maximum rate of LV pressure development and decline (dP/dt_max_ and dP/dt_min_), and the time constant of isovolumic relaxation (Tau, Weiss). Additionally, LV pressure-volume loops were recorded using conductance catheter technology. Following hemodynamic measurements, mice were euthanized and cardiac tissue was collected for further processing. For details on cardiac imaging and hemodynamic measurements, see File S1.

### Histological and Immunoblot Analysis

Ten weeks after TAC, hearts (without auricles) were weighed, and tibia length was measured to determine heart weight to body weight ratios (HW/BW) and heart weight to tibia length ratios (HW/TL). Myocardial PDE5 expression was examined using immunohistochemical stainings of 6 µm-thick LV sections. Furthermore, cardiac myocyte width was measured in laminin-stained sections, and to assess fibrosis in the LV, the area of collagen deposition and degree of collagen cross-links were traced on Sirius red-stained sections using polarized light. Apoptotic cardiac myocytes were detected by labeling DNA strand breaks using TUNEL (ApopTag, MilliPore), according to the manufacturer's instructions. In addition, immunoblot techniques were used to determine protein levels of PDE5, SERCA2, and markers of hypertrophy and oxidative stress. For detailed methods on immunohistochemistry and immunoblot analysis, see File S1.

### Quantitative Real-time Polymerase Chain Reaction and Cyclic Nucleotide Measurements

In mice with chronic pressure overload, myocardial expression of genes involved in cGMP metabolism, Ca^2+^-handling, cardiac hypertrophy, apoptosis, and fibrosis was measured using RT-qPCR (TaqMan or SYBR green PCR master mix, Life Technologies). For a list of specific primers see Table S1. Relative mRNA levels were analyzed using the Livak method [14].

To measure myocardial cyclic nucleotide concentrations, cyclic nucleotides were extracted from pulverized tissue in 6% trichloro-acetic acid, incubated at 4°C during 30 min, and centrifuged. Supernatant was extracted 3 times with water-saturated ether and vacuum-dried overnight. Lyophilized extracts were solubilized in assay buffer and cGMP and cAMP concentrations were measured using an enzyme immunoassay (GE Healthcare).

### Force Measurements in Isolated Permeabilized Cardiac Myocyte Preparations

Cardiac myocytes were isolated from pressure overloaded PDE5-TG and WT hearts, and permeabilized. In these cells, Ca^2+^-dependent active isometric force and its Ca^2+^-sensitivity, and Ca^2+^-independent passive force were determined as described previously [15], [16]. To determine cGMP/PKG-dependent modulation of the mechanical function of myofilaments, Ca^2+^-force relationships of cardiac myocytes were determined before and after incubation in relaxing solution supplemented with the catalytic subunit of bovine PKG, cGMP, and dithiothreitol. For details on force measurements, see File S1.

### Statistical Analysis

All data are expressed as mean±SEM. Differences between groups were determined with an unpaired t-test (with Welch's Correction) or a factorial ANOVA and a Bonferroni post hoc test. A probability value of *P*<0.05 was considered statistically significant.

## Results

### Increased PDE5 Expression in LV Tissue from Patients with Severe Aortic Stenosis

**Figure 1:**
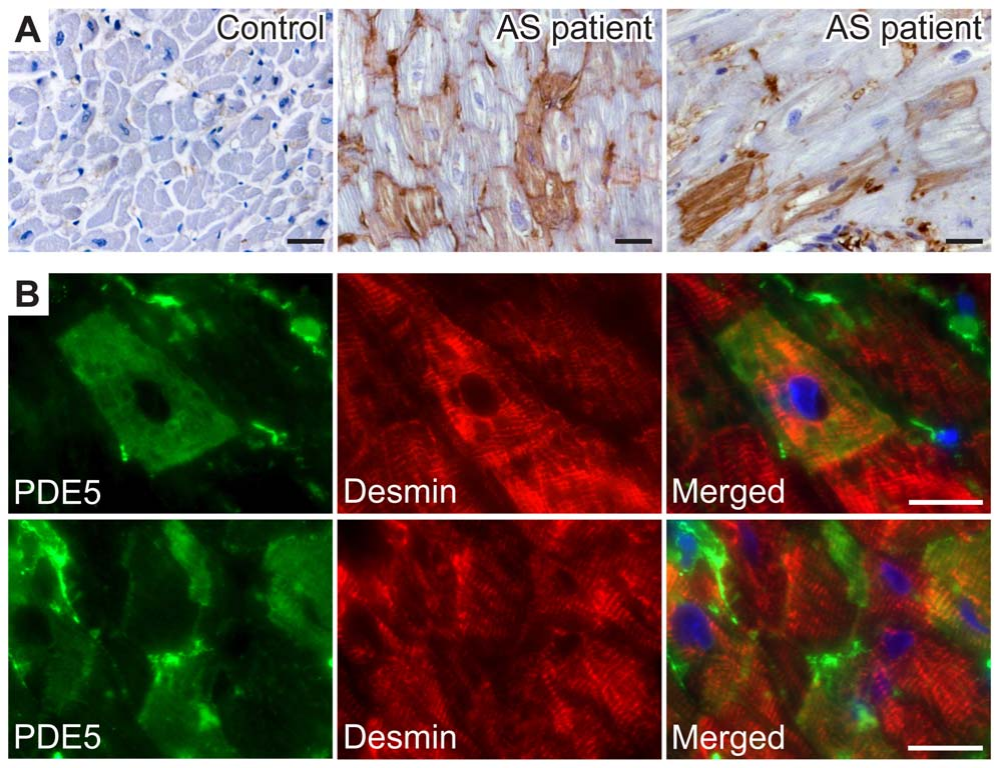
PDE5 expression in LV outflow tract tissue from patients with severe aortic stenosis. (**A**) In cardiac tissue of control subjects, PDE5 expression was limited. In contrast, in AS patients, marked PDE5 immunoreactivity was present in scattered cardiac myocytes. (**B**) Double immunofluorescent staining of PDE5 (green) and desmin (red) confirmed protein expression in cardiac myocytes. Scale bars, 25 µm.

Patients undergoing AVR (n = 20) had an aortic valve area ≤0.7 cm^2^, a transvalvular gradient of 57±3 mmHg, increased LV mass index of 86±4 g/m^2^, and no signs of obstructive coronary artery disease. To investigate spatial distribution of PDE5 in the heart, we performed immunohistochemical analyses of cardiac sections from control non-pressure overloaded hearts, obtained at autopsy (n = 5), and from AS patients. In cardiac tissue of control subjects, PDE5 expression was scarcely detectable (Figure 1A, left panel), and predominantly present in vascular smooth muscle cells. In contrast, in AS patients, PDE5 expression was markedly induced in scattered cardiac myocytes (Figure 1A and 1B). Histological examination of PDE5 immunoreactivity in cardiac tissue of AS patients was performed on a minimum of 10 high-power fields from 3 sections per patient and allowed a semi-quantitative scoring of PDE5 expression on a four-point scale from 0 to 3. Grade 0 indicated no PDE5 immunoreactivity in cardiac myocytes; grade 1, up to 1% PDE5 immunoreactive cardiac myocytes; grade 2, between 1 and 10% of PDE5 immunoreactive cardiac myocytes; and grade 3, PDE5 immunoreactivity of >10% of the cardiac myocytes. Subsequently, AS patients were separated into patients with grade 1 PDE5 expression, and those with markedly increased PDE5 immunoreactivity (grade 2–3). AS patients with grade 2–3 immunoreactivity (n = 13) had higher pulmonary capillary wedge pressures and serum NT-proBNP levels than AS patients with grade 1 PDE5 immunoreactivity (n = 7) (18±2 vs 11±1 mmHg, and 1090±254 vs 403±221 ng/l, respectively, *P*<0.05 for both).

### Cardiac PDE5 Expression is Increased in Mice in Response to Chronic Pressure Overload

**Figure 2:**
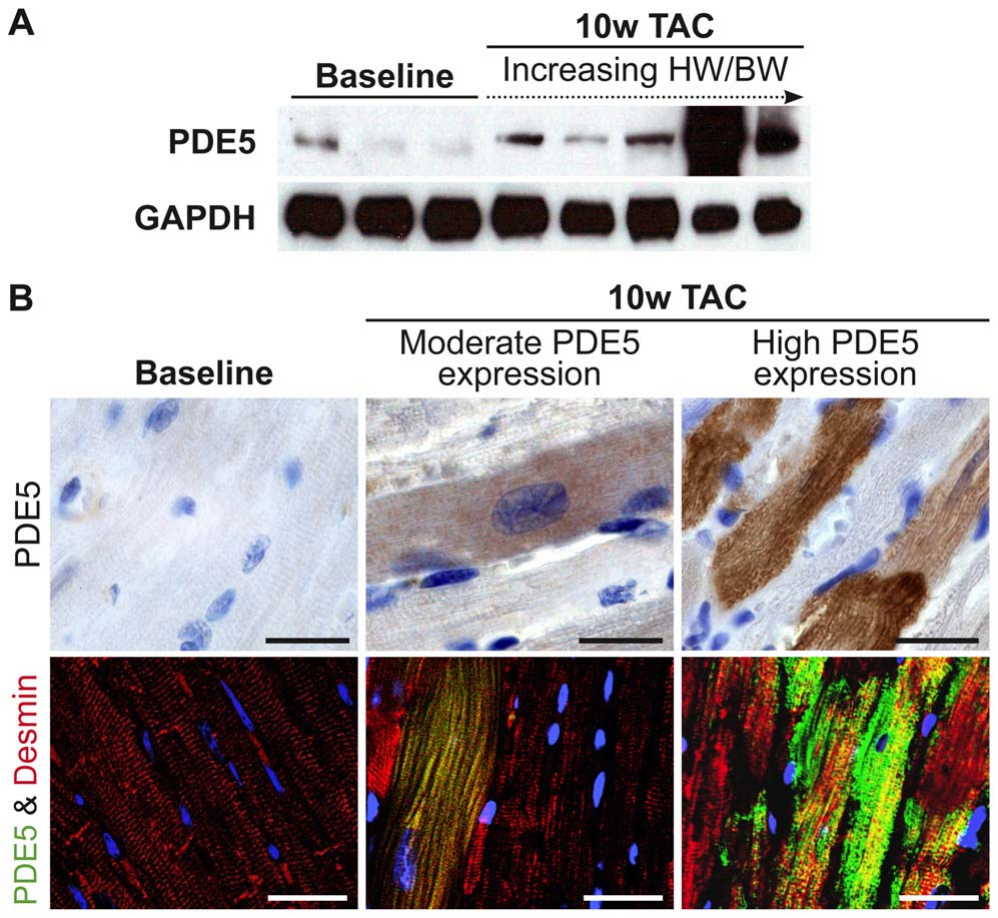
PDE5 expression in cardiac tissue from mice with chronic pressure overload. (**A**) Immunoblot analysis showed increased PDE5 protein expression after chronic pressure overload. Protein levels of GAPDH were measured to control for sample variability. (**B**) Immunohistochemical staining of PDE5 demonstrated that increased PDE5 expression was present in cardiac myocytes. Colocalization was confirmed in confocal images of double immunofluorescent staining of PDE5 (green) and desmin (red). Scale bars, 20 µm.

To investigate PDE5 expression in a mouse model of chronic pressure overload, we performed immunoblot and immunohistochemical analyses in WT mice at baseline and after 10 weeks TAC. Immunoblot analysis showed increased PDE5 protein expression after chronic pressure overload (Figure 2A). Histological examination of PDE5 immunoreactivity showed distinctly increased PDE5 expression in scattered cardiac myocytes (Figure 2B). A semi-quantitative scoring of PDE5 expression was performed on a four-point scale from 0 to 3 on a minimum of 20 high-power fields from 3 sections per mouse. Grade 0 indicated no PDE5 immunoreactivity in cardiac myocytes, grade 1; up to 1% of cardiac myocytes with distinctly increased PDE5 immunoreactivity, grade 2; marked PDE5 immunoreactivity in up to 50% of cardiac myocytes, and grade 3; robust immunoreactivity in >50% of the cardiac myocytes. In a second set of mice, PDE5 expression was quantified using PDE5 immunoblot and densitometric analysis. In mice with a modest increase in PDE5 protein levels (>1.5 fold versus baseline) and immunoreactivity (grade 1), we measured intermediate LV hypertrophy, and limited LV dysfunction and dilatation (Table 1). In contrast, mice with markedly increased PDE5 protein levels (>10-fold versus baseline) and immunoreactivity (grade 2–3), exhibited the most severe LV hypertrophy, LV dilatation, and impaired systolic function.

**Table 1:** Indices of cardiac remodeling after 10 weeks TAC in WT mice with increased PDE5 expression.

	Baseline	10 weeks TAC	
		Moderately increased PDE5 expression	Markedly increased PDE5 expression
	(n = 12)	(n = 11)	(n = 6)
**HW/BW** (mg/g)	3.5−4.2	6.3−9.0**^†^**	9.0−17.4**^†^** ^*^
**LVID_D_** (mm)	3.8±0.1	4.3±0.1**^†^**	5.4±0.2**^†^** ^*^
**EDV** (µl)	26±1	43±4**^†^**	85±8**^†^** ^*^
**LVID_S_** (mm)	2.7±0.1	3.7±0.1**^†^**	5.1±0.2**^†^** ^*^
**ESV** (µl)	14±1	28±4**^†^**	71±7**^†^** ^*^
**FS** (%)	27±2	15±2**^†^**	6±1**^†^** ^*^
**EF** (%)	46±3	37±3	17±2**^†^** ^*^
**HR** (bpm)	485±14	495±21	547±12

HW/BW indicates heart to body weight ratio; LVID_D_, LV internal diameter during diastole; EDV, end-diastolic volume; LVID_S_, LV internal diameter during systole; ESV, end-systolic volume; FS, fractional shortening; EF, ejection fraction; and HR, heart rate. **^†^** *P*<0.05 vs baseline, ^*^ *P*<0.05 vs mice with moderately increased PDE5 expression.

### PDE5 Overexpression in Cardiac Myocytes Worsens Cardiac Dysfunction and Adverse LV Remodeling in Mice after 10 weeks TAC

To investigate whether increased PDE5 expression in cardiac myocytes contributes to cardiac dysfunction and adverse LV remodeling in response to pressure overload or occurs secondary to the maladaptive cardiac response, we compared hemodynamics and LV remodeling in WT and PDE5-TG subjected to 10 weeks TAC.

Pressure-volume measurements immediately (i.e. within the first min) after TAC showed significantly and similarly increased end-systolic pressure (ESP), and an initial abrupt decrease in stroke volume (SV) and ejection fraction (EF), which remained similarly depressed in both genotypes during the first 15 minutes after TAC (Figure S1).

After 10 weeks TAC, WT sustained a higher pressure gradient across the fixed constriction in comparison to PDE5-TG, consistent with better preserved LV systolic function and maximum developed pressure in WT (Table 2). These findings were confirmed by load-independent conductance measurements, showing better preserved preload recruitable end-systolic stroke work in WT than in PDE5-TG (slope: 66.8±5.2 in 15 PDE5-TG vs 89.6±6.1 in 9 WT, *P*<0.05). Finally, indices of diastolic function were also impaired in PDE5-TG compared to WT (Table 2).

**Table 2:** Hemodynamic parameters in PDE5-TG and WT after 10 weeks TAC.

	10 weeks TAC	
	WT (n = 11)	PDE5-TG (n = 18)
**HW/BW** (mg/g)	7.2±0.5	6.8±0.2
**RCA-LCA gradient** (mmHg)	78±11	47±5^*^
**Maximum** **pressure** (mmHg)	145±7	106±5^*^
**dP/dt_max_** (mmHg/s)	9811±809	5868±375^*^
**dP/dt_min_** (mmHg/s)	−9638±769	−5989±453^*^
**Tau** (ms)	10.9±1.1	13.6±1.0^*^
**HR** (bpm)	580±29	519±22

HW/BW indicates heart to body weight ratio; RCA-LCA gradient, gradient between right and left common carotid artery; dP/dt_max_ and dP/dt_min_, maximum and minimum of the first derivative of LV pressure over time; Tau, time constant for isovolumic relaxation (Weiss); and HR, heart rate. *P<0.05 vs WT.

**Figure 3:**
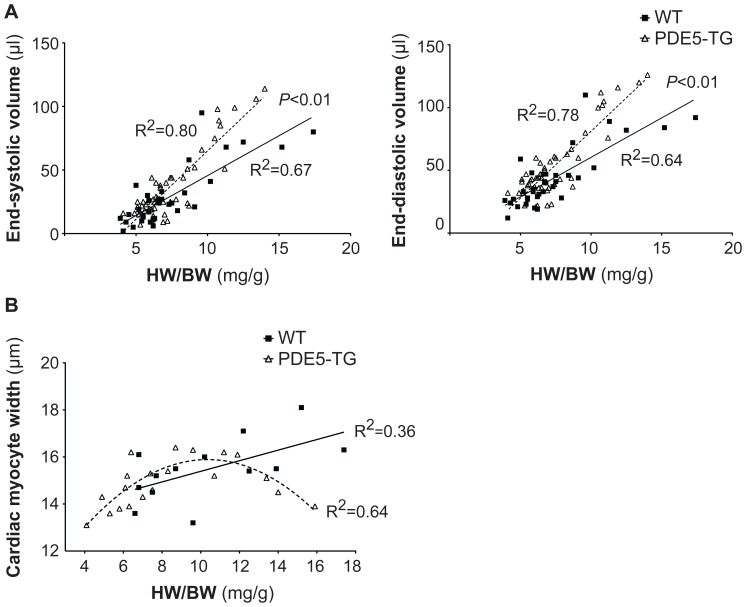
LV volumes and cardiac myocyte width in PDE5-TG and WT after 10 weeks TAC. (**A**) In mice with high HW/BW, TTE revealed greater end-systolic and end-diastolic LV volumes in PDE5-TG (n = 49) than in WT (n = 35), resulting in a significantly steeper slope of the regression curve, and suggesting greater propensity to LV dilatation in PDE5-TG. (**B**) Moreover, unlike in WT (n = 13), cardiac myocyte width in PDE5-TG (n = 21) did not augment linearly with increasing HW/BW, consistent with a more dilated phenotype.

In addition, TTE revealed greater LV dimensions in PDE5-TG than in WT (LVID_S_: 4.0±0.1 vs 3.6±0.2 mm and LVID_D_ 4.6±0.1 vs 4.2±0.1 mm, in 49 PDE5-TG vs 35 WT respectively, both *P*<0.05). In PDE5-TG, the slope of the regression curves between increasing HW/BW and end-systolic and end-diastolic volumes was significantly different than in WT, suggesting greater adverse remodeling with increasing hypertrophic stress (Figure 3A). Moreover, cardiac myocyte width, measured on laminin-stained tissue sections, increased proportionately with HW/BW in WT, whereas PDE5-TG showed reduced cardiac myocyte width with high HW/BW, consistent with a more dilated phenotype (Figure 3B).

### Increased Cardiac Myocyte-specific PDE5 Expression does not Affect Myocardial Hypertrophy and Extracellular Matrix Remodeling in Response to Sustained Pressure Overload

Ten weeks after TAC, HW/BW and HW/TL increased significantly and similarly in PDE5-TG and WT, compared to baseline (Table S2). Furthermore, fetal gene expression was similar in PDE5-TG and WT (Table S2), as well as levels of proteins known to be markers of hypertrophy (Figure S2A). Deposition of thick, tightly-packed red birefringent collagen fibers and thin, loosely-assembled green birefringent collagen fibers (Figure S2B), and fibronectin content were increased to the same extent in the two genotypes, consistent with similar levels of mRNAs encoding transforming growth factor (TGF)-β1 and connective tissue growth factor (CTGF; Table S3). There was also no difference in markers of apoptosis and oxidative stress between the two genotypes (Figure S2B and S2C, and Table S3). Taken together, these data indicate that LV dysfunction in PDE5-TG cannot be explained by cardiac hypertrophy, fibrosis, apoptosis, or oxidative stress in response to sustained pressure overload.

### Enhanced Cardiac Myocyte-specific PDE5 Expression Limits the Increase in Myocardial cGMP Levels in Response to Chronic Pressure Overload

Ten weeks after TAC, myocardial cGMP levels were lower in PDE5-TG (0.05±0.01 pmol/mg protein, n = 26) than in WT (0.11±0.02 pmol/mg protein, n = 15, *P*<0.05). This reduction was not attributable to altered expression levels of genes encoding soluble and particulate guanylate cyclases (sGC and pGC), and other PDE isoforms (data not shown). In addition, myocardial levels of cAMP, another important second messenger in the heart, were similar in both genotypes after 10 weeks TAC (4.2±0.6 in PDE5-TG vs 3.5±0.4 pmol/mg protein in WT, *P* = NS).

### PDE5 Overexpression is Associated with Decreased SERCA2 Expression and Increased Passive Force of Cardiac Myocytes in Pressure Overloaded Hearts

To investigate the molecular mechanisms of greater contractile dysfunction in PDE5-TG after 10 weeks TAC, we measured cardiac levels of the sarcoplasmic reticulum (SR) Ca^2+^-ATPase 2 SERCA2, and active and passive forces in isolated cardiac myocytes.

**Figure 4:**
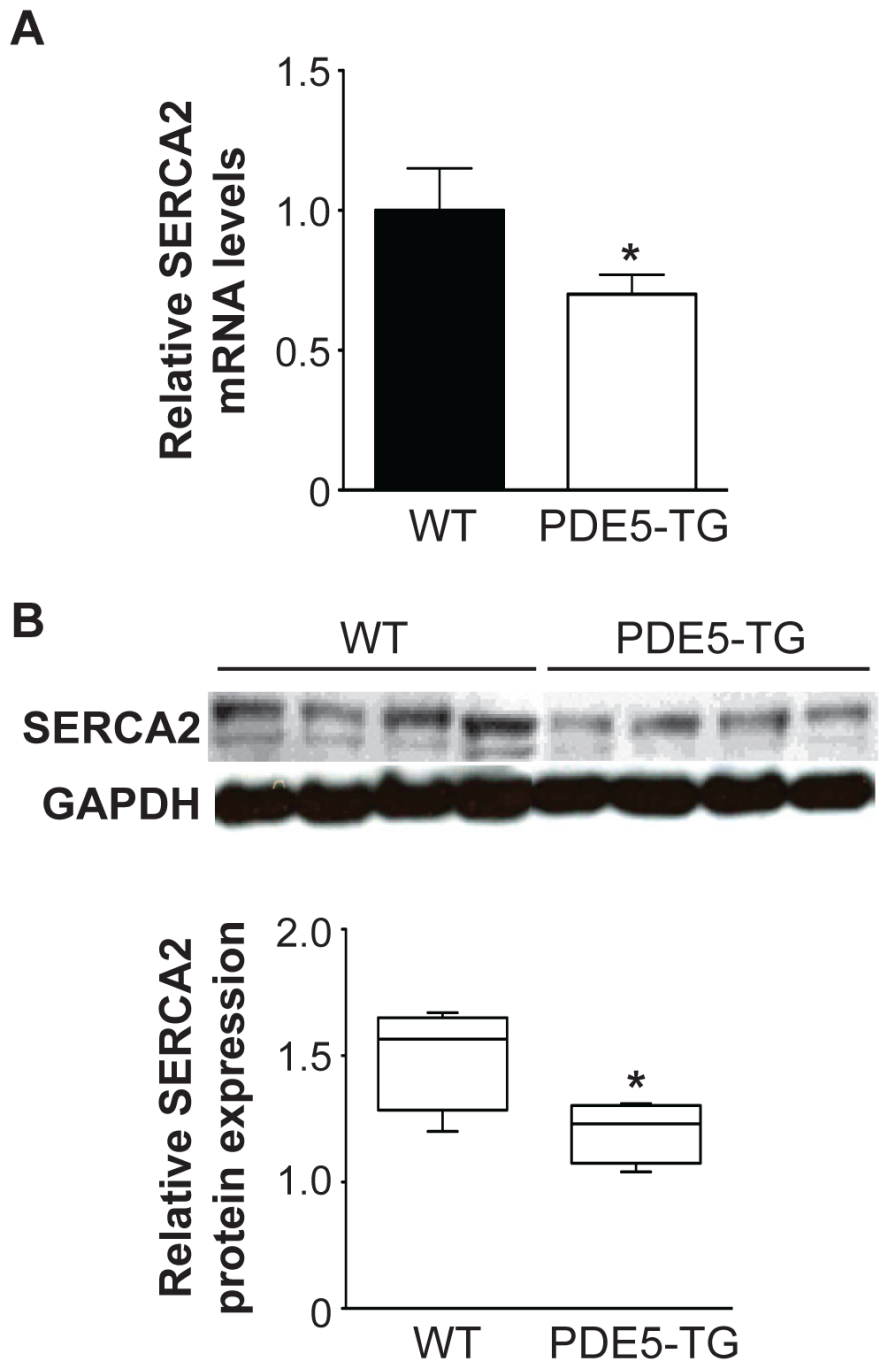
Myocardial levels of SERCA2 in PDE5-TG and WT after 10 weeks TAC. Transcript levels (**A**) and protein levels (**B**) of SERCA2, measured using RT-qPCR and immunoblot and densitometric analysis, respectively, were significantly lower in PDE5-TG than in WT. Transcript and protein levels of GAPDH were measured for normalization. ^*^ *P*<0.05 vs WT.

Quantitative PCR and immunoblot analysis showed lower SERCA2 gene and protein expression in PDE5-TG than in WT (*P*<0.05, Figure 4A and 4B), consistent with more pronounced abnormal Ca^2+^-handling and contractile impairment [17].

**Figure 5:**
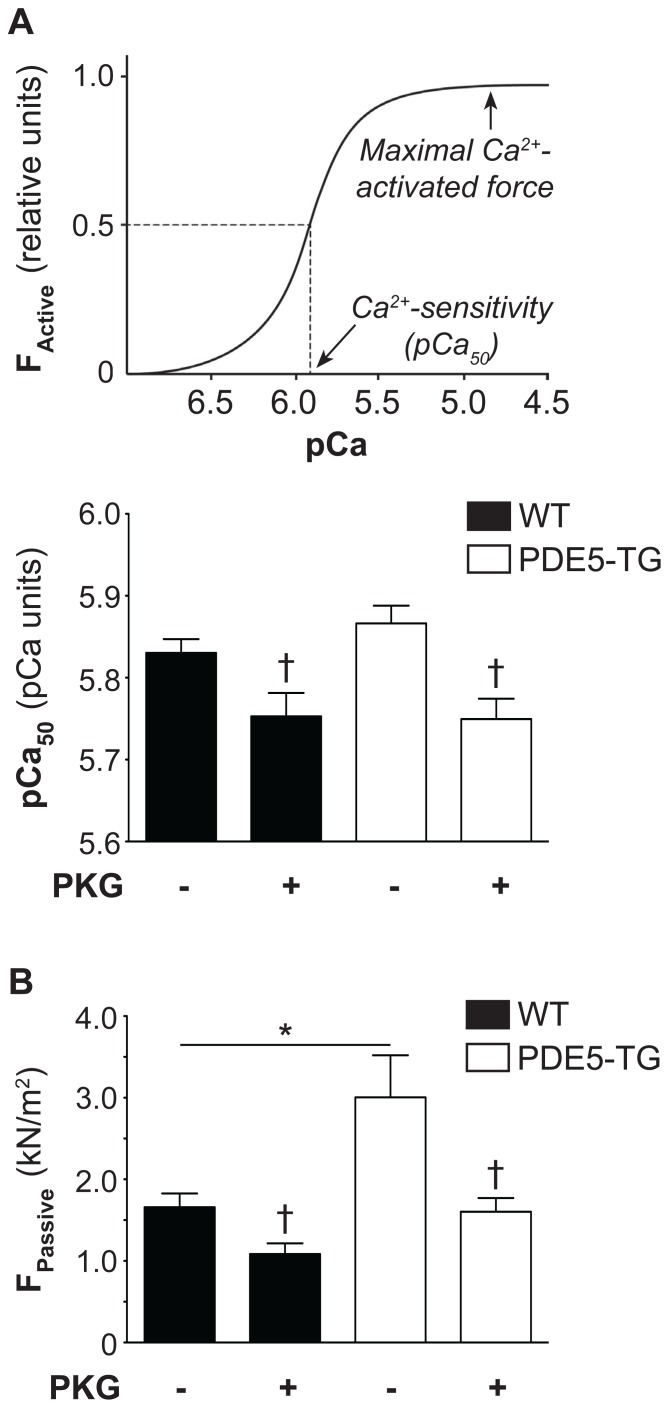
Ca^2+^-sensitivity of active force, and passive force in PDE5-TG and WT cardiac myocytes after chronic pressure overload. (**A**) In the Ca^2+^-force relationship, the Ca^2+^-sensitivity of isometric force production represents the [Ca^2+^] (plotted as a function of pCa; pCa = −log_10_[Ca^2+^]) evoking half-maximal force production (*top panel*). Ca^2+^-sensitivity did not differ between PDE5-TG and WT cardiac myocytes, and was reduced after pretreatment with PKG (*bottom panel*). (**B**) Passive forces were significantly greater in cardiac myocytes from PDE5-TG compared to WT cardiac myocytes. Pretreatment of cardiac myocytes with PKG significantly reduced the passive forces in both genotypes. *P<0.05; ^†^ *P*<0.05 vs without PKG.

Active forces of permeabilized cardiac myocytes from 5 PDE5-TG (HW/BW = 8.5±0.8 mg/g, 20 cells) and 4 WT (HW/BW = 8.5±2.5 mg/g, 16 cells) did not differ (13.9±1.7 vs 11.3±1.8 kN/m^2^, *P* = NS). Ca^2+^-sensitivity of isometric force production, determined as the [Ca^2+^] at which 50% of maximal Ca^2+^-activated force was reached (Figure 5A, top panel), was not different between genotypes, and was equally decreased after pretreatment with cGMP-dependent PKG-Iα (Figure 5A, bottom panel). In contrast, passive forces were significantly higher in PDE5-TG than in WT, consistent with more pronounced diastolic dysfunction in PDE5-TG. Passive forces were again reduced after pretreatment of cardiac myocytes with cGMP-dependent PKG-Iα in both genotypes (Figure 5B).

## Discussion

In this study we report elevated myocardial PDE5 expression in patients with severe aortic stenosis undergoing AVR, and in mice exposed to chronic pressure overload induced by TAC. We detected a strikingly similar PDE5 expression pattern in cardiac myocytes of human and murine hearts subjected to pronounced hypertrophic stress resulting in marked LV dysfunction. To determine whether increased PDE5 levels in cardiac myocytes contribute to the adverse LV remodeling in response to increased load, we took advantage of transgenic mice with cardiac myocyte-specific PDE5 overexpression [8]. These mice display normal hemodynamics at baseline but develop greater adverse LV remodeling and cardiac dysfunction than WT littermates after 10 weeks TAC. Transgenic mice had a blunted myocardial cGMP response to chronic pressure overload, lower cardiac levels of the SR Ca^2+^-ATPase SERCA2 and greater PKG-Iα-sensitive increases in passive force of permeabilized cardiac myocytes. Taken together, our data suggest that in the context of chronic increased afterload, PDE5 induction in cardiac myocytes impairs contractile function, increases myocardial passive stiffness, and is associated with adverse LV remodeling.

*In vivo* animal studies have provided compelling evidence for a protective role of cGMP signaling during various stress conditions leading to deleterious LV remodeling [18]. Enhanced PDE5 expression has been associated with right ventricular hypertrophy and heart failure in patients [6], [7], [8]. The most striking observation in our study is a patchy PDE5 cardiac myocyte expression pattern in those AS patients with clear signs of heart failure (i.e. higher LV filling pressures and circulating BNP levels), and in mice with marked adverse cardiac remodeling and dysfunction induced by chronic pressure overload. The role of increased PDE5 in pressure overloaded hearts has been studied using small molecule inhibitors of cGMP hydrolysis. Multiple reports have shown a beneficial effect of sildenafil against adverse structural and functional cardiac remodeling in mice, and very recently also on hemodynamics in a small-scale intervention study in severe AS patients [19]. To what extent beneficial effects of sildenafil on cardiac remodeling involve targeting of PDE1 [20], non-myocyte cardiac cell types, and indirect modulation of LV afterload via vasodilatation of systemic resistance vessels, remains incompletely understood. Our observations of prominent LV dilatation and dysfunction in mice with transgenic overexpression of PDE5 in cardiac myocytes, suggest a key role for cardiac myocyte PDE5 in adverse remodeling in chronic pressure overloaded hearts.

In a recent study, 6 weeks of TAC induced concentric and generally compensated hypertrophy in WT, but pathological hypertrophy and amplified fibrosis with signs of heart failure in mice with conditional cardiac myocyte-specific PDE5 overexpression [21]. Tetracycline-induced reversal of transgene expression or sildenafil administration after 1 week TAC abrogated this maladaptive remodeling, and ameliorated LV dilatation and dysfunction. In our study, the degree of cardiac hypertrophy and fibrosis was not affected in PDE5-TG after 10 weeks TAC, possibly related to the different duration of TAC, the life-long versus inducible overexpression of the PDE5 transgene, and the mouse strain used [22].

The reasons for impaired LV function and remodeling in PDE5-TG may be multiple. First, lower mRNA and protein levels of SERCA2 are observed in transgenic mice after 10 weeks TAC. Impaired Ca^2+^-handling associated with reduced SERCA2 expression and activity is the molecular hallmark of heart failure, and a recent target for therapeutic interventions [23]. Reduced SERCA2 expression indirectly indicates decreased Ca^2+^-transients in these transgenic mice, heralding systolic dysfunction. However, this remains to be empirically determined via whole-cell patch clamping.

Second, myocardial cGMP levels increased in WT but not in PDE5-TG subjected to sustained pressure overload. These reduced cGMP levels in pressure overloaded PDE5-TG hearts may result in diminished PKG-dependent phosphorylation of the cardiac-specific N2B element of titin, an extensible molecular spring in stretched sarcomeres [24]. PKG-mediated titin phosphorylation causes reduction of passive forces in cardiac myocytes, thereby representing a potential mechanism for increased passive forces in PDE5-TG cardiac myocytes, and thus greater myocardial stiffness and diastolic dysfunction in PDE5-TG. The observed *in vitro* cGMP/PKG-dependent reduction of passive force is consistent with this post-translational modulation, although changes in titin isoform expression can also modulate passive force of cardiac myocytes [25].

In conclusion, PDE5 expression is markedly increased in cardiac myocytes of aortic stenosis patients with signs of heart failure and mice with prominent adverse LV remodeling and dysfunction in response to chronic pressure overload. Cardiac myocyte-restricted overexpression of PDE5 impairs contractile function and LV remodeling in pressure overloaded murine hearts, and is associated with reduced SERCA2 levels and increased passive cardiac myocyte forces. Increased cardiac myocyte PDE5 expression represents a molecular hallmark during chronic pressure overload, heralding transition to adverse LV remodeling and dysfunction, and a future target for therapeutic intervention.

## Supporting Information

Figure S1 **Hemodynamic parameters in PDE5-TG and WT during the first fifteen minutes after aortic constriction.** (**A**) In PDE5-TG (n = 9) and WT (n = 7), an instantaneous increase in end-systolic pressure is observed upon aortic banding, followed by a progressive decrease over the following 15 minutes. (**B**) Simultaneously, an abrupt and equal decrease in stroke volume and ejection fraction is observed immediately after banding in both genotypes.
(TIF)

Figure S2 **Structural LV remodeling in pressure overloaded PDE5-TG and WT hearts.** (**A**) Protein levels of several components of the cardiac hypertrophy pathway were similar in both genotypes after 10 weeks TAC. GAPDH protein levels were measured to control for sample variability. (**B**) Cardiac fibrosis and apoptosis were also comparable in pressure overloaded PDE5-TG and WT. To assess the degree of fibrosis in the murine LV, the area of collagen deposition was traced on Sirius red-stained tissue sections using polarized light, allowing evaluation of tightly-packed red birefringent collagen and thin, loosely-assembled green birefringent collagen. Cardiac myocyte apoptosis was evaluated by labeling and detecting DNA strand breaks (TUNEL). (**C**) Myocardial levels of the oxidative stress markers 3-nitrotyrosine, 4-hydroxy-2-nonenal, and malondialdehyde were similar in PDE5-TG and WT with sustained pressure overload. GAPDH protein levels were measured to control for sample variability. Scale bars = 50 µm.
(TIF)

Table S1 **Sequences of primers and probes used for quantitative real-time PCR.** ANP and BNP indicate atrial and brain natriuretic peptide; Bax, Bcl-2 associated X protein; Bcl-2 and Bcl-X_L_, B-cell lymphoma 2 and extra large; CTGF, connective tissue growth factor; Fas and FasL, Fas receptor and ligand; FN, fibronectin; GAPDH, glyceraldehyde-3′-phosphate-dehydrogenase; PDE5, phosphodiesterase type 5; SERCA2, sarcoplasmic reticulum Ca^2+^-ATPase 2; and TGF-β1, transforming growth factor-β1.
(DOC)

Table S2 **Indices of cardiac hypertrophy in PDE5-TG and WT after 10 weeks TAC.** HW/BW indicates heart weight to body weight ratio; HW/TL, heart weight to tibia length ratio; and ANP and BNP, atrial and brain natriuretic peptide. ^†^P<0.05 vs baseline.
(DOCX)

Table S3 **Indices of cardiac fibrosis and apoptosis in PDE5-TG and WT after 10 weeks TAC.** FN indicates fibronectin; TGF-β1, transforming growth factor-β1; CTGF, connective tissue growth factor; Bcl-2 and Bcl-X_L_, B-cell lymphoma 2 and extra large; Bax, Bcl-2 associated X protein; and Fas and FasL, Fas receptor and ligand.
(DOCX)

File S1 **Supporting Materials and Methods.**
(DOC)
