# Supplementary figures and images for: Increased Cardiac Myocyte PDE5 Levels in Human and Murine Pressure Overload Hypertrophy Contribute to Adverse LV Remodeling
Source: PLoS One. 2013 Mar 18;8(3):e58841. doi: 10.1371/journal.pone.0058841 (PMC3601083; doi:10.1371/journal.pone.0058841)

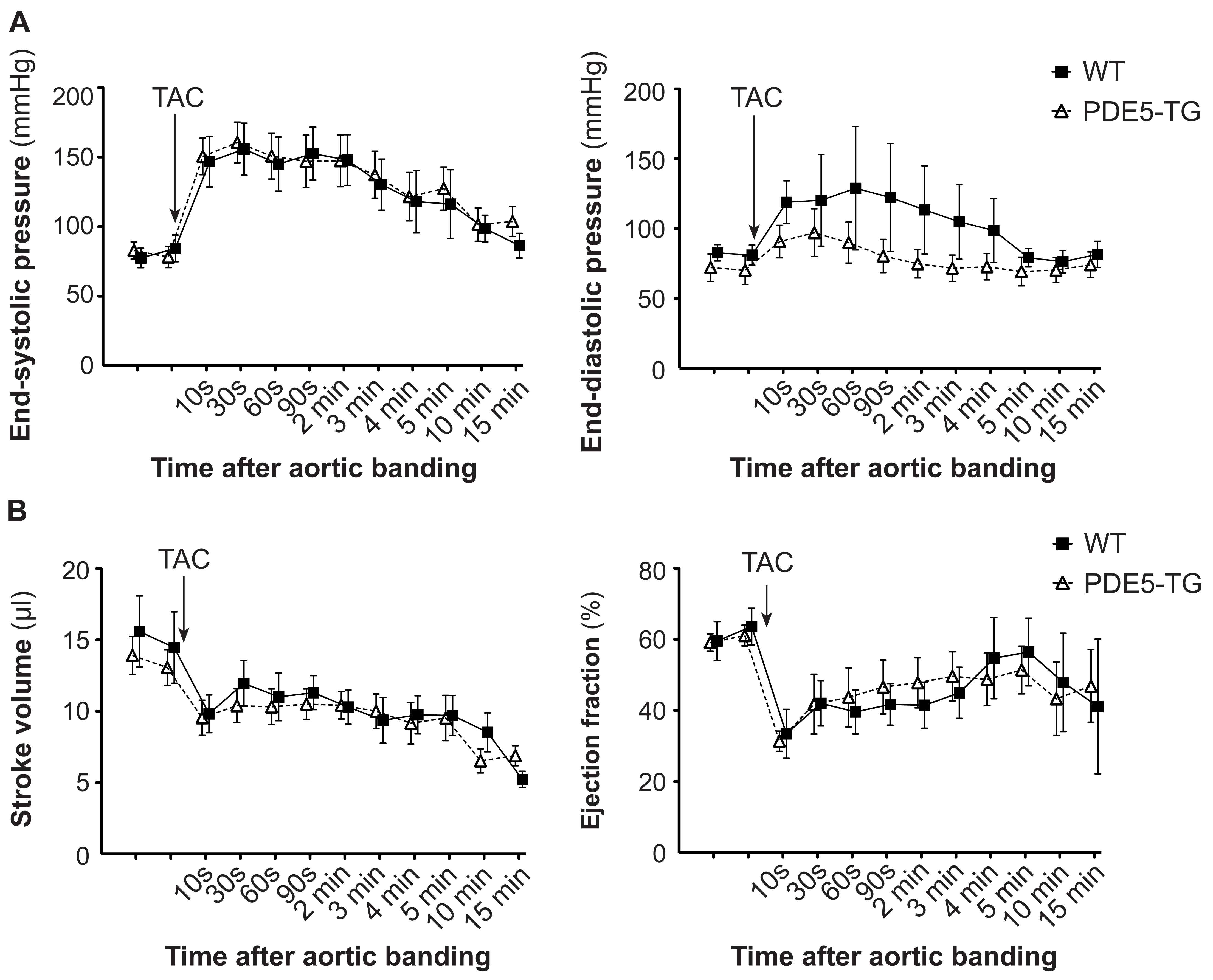

Supplement: Figure S1 — Hemodynamic parameters in PDE5-TG and WT during the first fifteen minutes after aortic constriction. (A) In PDE5-TG (n = 9) and WT (n = 7), an instantaneous increase in end-systolic pressure is observed upon aortic banding, followed by a progressive decrease over the following 15 minutes. (B) Simultaneously, an abrupt and equal decrease in stroke volume and ejection fraction is observed immediately after banding in both genotypes. (TIF) [file pone.0058841.s001.tif]

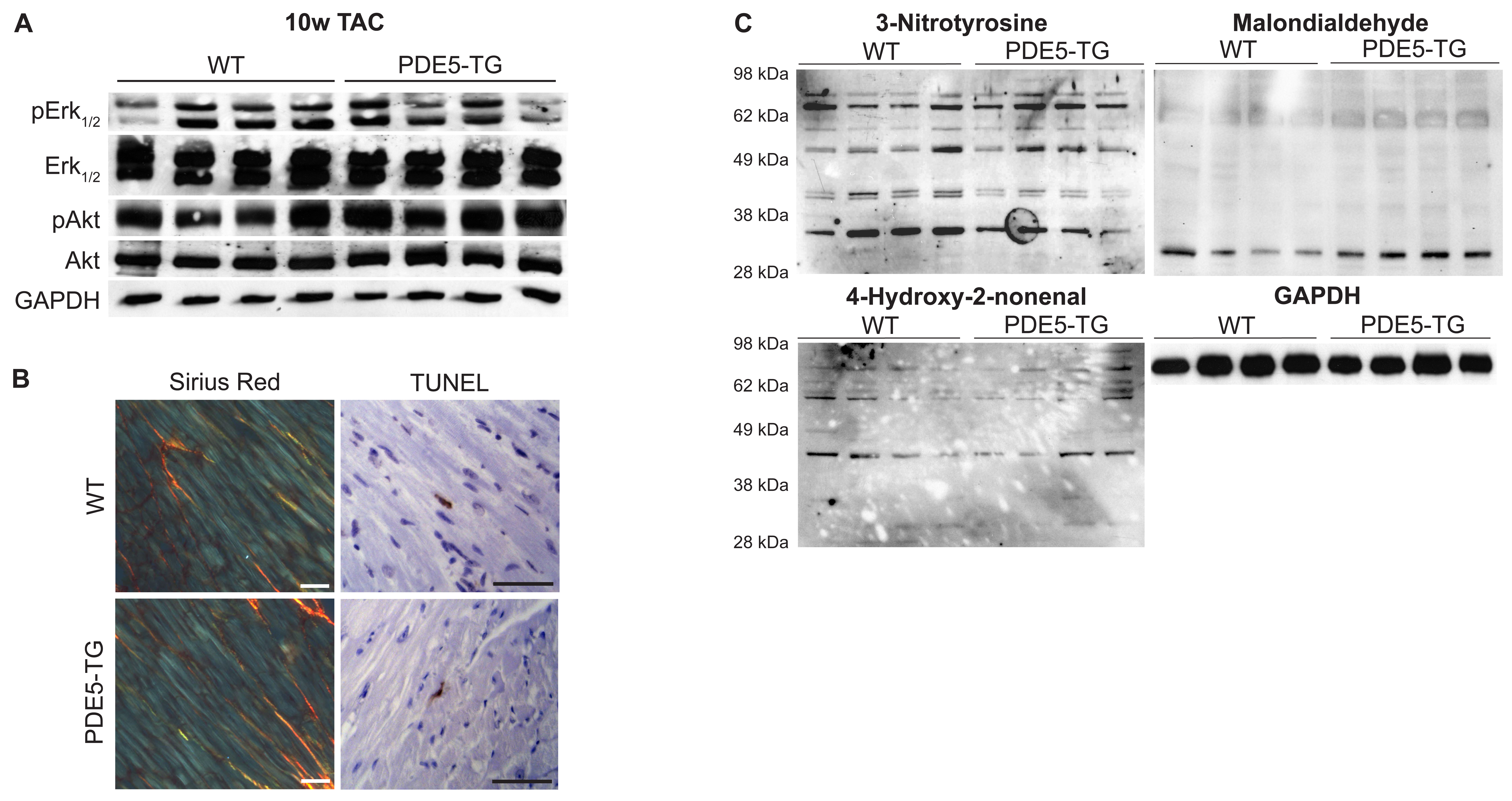

Supplement: Figure S2 — Structural LV remodeling in pressure overloaded PDE5-TG and WT hearts. (A) Protein levels of several components of the cardiac hypertrophy pathway were similar in both genotypes after 10 weeks TAC. GAPDH protein levels were measured to control for sample variability. (B) Cardiac fibrosis and apoptosis were also comparable in pressure overloaded PDE5-TG and WT. To assess the degree of fibrosis in the murine LV, the area of collagen deposition was traced on Sirius red-stained tissue sections using polarized light, allowing evaluation of tightly-packed red birefringent collagen and thin, loosely-assembled green birefringent collagen. Cardiac myocyte apoptosis was evaluated by labeling and detecting DNA strand breaks (TUNEL). (C) Myocardial levels of the oxidative stress markers 3-nitrotyrosine, 4-hydroxy-2-nonenal, and malondialdehyde were similar in PDE5-TG and WT with sustained pressure overload. GAPDH protein levels were measured to control for sample variability. Scale bars = 50 µm. (TIF) [file pone.0058841.s002.tif]
