# Supplementary material for: Increased Cardiac Myocyte PDE5 Levels in Human and Murine Pressure Overload Hypertrophy Contribute to Adverse LV Remodeling
Source: PLoS One. 2013 Mar 18;8(3):e58841. doi: 10.1371/journal.pone.0058841 (PMC3601083; doi:10.1371/journal.pone.0058841)
Supplement: Table S1 — Sequences of primers and probes used for quantitative real-time PCR. ANP and BNP indicate atrial and brain natriuretic peptide; Bax, Bcl-2 associated X protein; Bcl-2 and Bcl-XL, B-cell lymphoma 2 and extra large; CTGF, connective tissue growth factor; Fas and FasL, Fas receptor and ligand; FN, fibronectin; GAPDH, glyceraldehyde-3′-phosphate-dehydrogenase; PDE5, phosphodiesterase type 5; SERCA2, sarcoplasmic reticulum Ca2+-ATPase 2; and TGF-β1, transforming growth factor-β1. (DOC) [file pone.0058841.s003.doc]

**Table S1.** **Sequences of primers and probes used for quantitative real-time PCR.**

| ***Mouse*** | **Forward primer** | **Reverse primer** | **Probe** |
| --- | --- | --- | --- |
| **ANP** | 5’-TCCATCACCCTGGGCTTCT-3’ | 5’-AGCATTTGGTCCAATATGGCC -3’ | 5’-CCTCGTCTTGGCCTTTTGGCTTCC-3’ |
| **Bax** | 5’-CCGGCGAATTGGAGATGA-3’ | 5’-CCCAGTTGAAGTTGCCATCA-3’ | 5’- TGGACACGGACTCCCCCCGA-3’ |
| **BNP** | 5’-GCCAGTCTCCAGAGCAATTCA-3’ | 5’-GTGAGGCCTTGGTCCTTCAA-3’ | 5’-TCTGGGCCATTTCCTCCGACTTTTCT-3’ |
| **Bcl-2** | 5’-TGGCCTTCTTTGAGTTCGGT-3’ | 5’-GAGAAATCAAACAGAGGTCGCAT-3’ | - |
| **Bcl-X_L_** | 5’-ACTCATCGCCTGCCTCTCTC-3’ | 5-GCCACAGCAGCAGTTTGGAT-3’ | - |
| **CTGF** | 5’-TGACCCCTGCGACCCACA-3’ | 5’-TACACCGACCCACCGAAGACACAG-3’ | - |
| **Fas** | 5’-TAGAACCTCCAGTCGTGAAACCATA-3’ | 5’-TTTAGCTTCCTGGATTGTCATGTC-3’ | - |
| **FasL** | 5’-AGTGTCTCATTGGCACCATCTTTA-3’ | 5’-CAAACATCCCTCTTACTTCTCCGTTA-3’ | - |
| **FN** | 5’-CCGGTGGCTGTCAGTCAGA-3’ | 5’-CCGTTCCCACTGCTGATTTATC-3’ | - |
| **GAPDH** | 5’-TGTGTCCGTCGTGGATCTGA-3’ | 5’-CCTGCTTCACCACCTTCTTGA-3’ | 5’-CCTGGAGAAACCTGCAAGTATGATGACA-3’ |
| **PDE5** | 5’-CGGCCTACCTGGCATTCT G-3’ | 5’-GCAAGGTCAAGTAACACCTGATT-3’ | - |
| **SERCA2** | 5’-CATCTGCTTGTCCATGTCACTT-3’ | 5’- CGGTGTGATCTGGAAAATGAG -3’ | 5’-TCTTGATCCTCTACGTGGAACCTTTGC-3’ |
| **TGF-β1** | 5’-GACCCTGCCCCTATATTTGGA-3’ | 5’-GCGCCCGGGTTGTGT-3’ | - |
