# Supplementary material for: Increased Cardiac Myocyte PDE5 Levels in Human and Murine Pressure Overload Hypertrophy Contribute to Adverse LV Remodeling
Source: PLoS One. 2013 Mar 18;8(3):e58841. doi: 10.1371/journal.pone.0058841 (PMC3601083; doi:10.1371/journal.pone.0058841)
Supplement: Table S2 — Indices of cardiac hypertrophy in PDE5-TG and WT after 10 weeks TAC. HW/BW indicates heart weight to body weight ratio; HW/TL, heart weight to tibia length ratio; and ANP and BNP, atrial and brain natriuretic peptide. †P<0.05 vs baseline. (DOCX) [file pone.0058841.s004.docx]

|  | **Baseline** | |
| --- | --- | --- |
|  | **WT** | **PDE5-TG** |
| **HW/BW** (mg/g) | 4.0±0.1 (n=14) | 3.9±0.1 (n=20) |
| **HW/TL** (mg/cm) | 5.9±0.1 (n=14) | 6.3±0.1 (n=20) |
|  | **10 weeks TAC** | |
|  | **WT** | **PDE5-TG** |
| **HW/BW** (mg/g) | 7.6±0.3**^†^** (n=38) | 7.8±0.5**^†^** (n=52) |
| **HW/TL** (mg/cm) | 119.8±4.0**^†^** (n=38) | 115.5±5.5**^†^** (n=52) |
| **ANP** mRNA levels | 1.00±0.40 (n=8) | 1.76±0.43 (n=16) |
| **BNP** mRNA levels | 1.00±0.40 (n=8) | 0.87±0.22 (n=16) |

**Table S2. Indices of cardiac hypertrophy in PDE5-TG and WT after 10 weeks TAC.**
