# Supplementary material for: Increased Cardiac Myocyte PDE5 Levels in Human and Murine Pressure Overload Hypertrophy Contribute to Adverse LV Remodeling
Source: PLoS One. 2013 Mar 18;8(3):e58841. doi: 10.1371/journal.pone.0058841 (PMC3601083; doi:10.1371/journal.pone.0058841)
Supplement: Table S3 — Indices of cardiac fibrosis and apoptosis in PDE5-TG and WT after 10 weeks TAC. FN indicates fibronectin; TGF-β1, transforming growth factor-β1; CTGF, connective tissue growth factor; Bcl-2 and Bcl-XL, B-cell lymphoma 2 and extra large; Bax, Bcl-2 associated X protein; and Fas and FasL, Fas receptor and ligand. (DOCX) [file pone.0058841.s005.docx]

**Table S3. Indices of cardiac fibrosis and apoptosis in PDE5-TG and WT after 10 weeks TAC.**

|  | **10 weeks TAC** | |
| --- | --- | --- |
|  | **WT** | **PDE5-TG** |
| **Red birefringent collagen** (% tissue area) | 4.7±0.8 (n=5) | 4.2±1.1 (n=5) |
| **Green birefringent collagen** (% tissue area) | 1.3±0.4 (n=5) | 1.5±0.7 (n=5) |
| **FN** mRNA levels | 1.00±0.30 (n=13) | 0.79±0.18 (n=19) |
| **TGF-β1** mRNA levels | 1.00±0.32 (n=13) | 0.64±0.27 (n=19) |
| **CTGF** mRNA levels | 1.00±0.25 (n=13) | 1.05±0.28 (n=19) |
| **# Apoptotic cardiac myocytes** (/ 10 mm^2^) | 13±4 (n=5) | 18±4 (n=7) |
| **Bcl-2** mRNA levels | 1.00±0.24 (n=12) | 2.22±0.79 (n=19) |
| **Bcl-X_L_** mRNA levels | 1.00±0.48 (n=8) | 0.76±0.18 (n=11) |
| **Bax** mRNA levels | 1.00±0.23 (n=12) | 1.98±0.75 (n=18) |
| **Fas** mRNA levels | 1.00±0.29 (n=13) | 0.46±0.14 (n=21) |
| **FasL** mRNA levels | 1.00±0.43 (n=13) | 0.30±0.12 (n=21) |
