# Supplementary material for: Increased Cardiac Myocyte PDE5 Levels in Human and Murine Pressure Overload Hypertrophy Contribute to Adverse LV Remodeling
Source: PLoS One. 2013 Mar 18;8(3):e58841. doi: 10.1371/journal.pone.0058841 (PMC3601083; doi:10.1371/journal.pone.0058841)
Supplement: File S1 — Supporting Materials and Methods. (DOC) [file pone.0058841.s006.doc]

**Increased Cardiac Myocyte PDE5 Levels in Human and Murine Pressure Overload Hypertrophy Contribute to Adverse LV Remodeling**

Vandenwijngaert S., Pokreisz P., Hermans H., Gillijns H., Pellens M., Bax N.A.M., Coppiello G., Oosterlinck W., Balogh A., Papp Z., Bouten C.V.C., Bartunek J., D'hooge J., Luttun A., Verbeken E., Herregods M.C., Herijgers P., Bloch K.D., and Janssens S.

# Supporting File S1: Supporting Materials and Methods

**Transverse Aortic Constriction**

For TAC surgeries, mice were anesthetized with sodium pentobarbital (Ceva Santé Animale, 40-70 mg/kg IP), and ventilated at 150 breaths per minute (0.25 ml tidal volume, MiniVent, Harvard Apparatus). The body temperature was monitored and maintained at 37°C using a rectal probe and heating pad (TC-1000, CWE Inc.). For banding of the aorta, the chest cavity was opened, and a curved forceps was placed under the transverse aorta. Next, a 7-0 silk suture was grasped by the forceps and moved underneath the aorta, and a loose double knot was made. A 27-gauge needle was delivered through the loose knot and placed parallel to the aorta. The loop was then tied around the aorta and needle, and secured with the second knot. The needle was immediately removed, leaving a discrete region of stenosis. Depth of anesthesia was controlled by monitoring muscle tone (toe pinch) during the entire procedure. Following TAC, all layers of muscle and skin were closed using a 6.0 ticron suture, the wound was treated with an antiseptic, and an analgesic (buprenorphine, Schering-Plough, 0.1 mg/kg SC) was administered during the first two days.

**Transthoracic Echocardiography**

After 10 weeks TAC, mice were sedated with 1.5% isoflurane (Ecuphar) and standard views were obtained in two-dimensional and M-mode using transthoracic echocardiography with a 13-MHz transducer (i13L) on a GE Vivid7 (GE Healthcare) echocardiograph. Body temperature was monitored and maintained at 37°C using a rectal probe and heating pad (TC-1000, CWE Inc.).

**Hemodynamic Measurements**

For invasive *pressure measurements*, mice were anesthetized with urethane (Sigma-Aldrich, 1.2-1.4 g/kg, IP) and etomidate (10 mg/kg, IP), spontaneously breathing, and temperature was monitored and controlled using a rectal probe and heating pad (TC-1000, CWE Inc.). Right and left common carotid arteries were exposed, and a 1.4-F high fidelity pressure-conductance catheter (Millar Instruments) was first inserted into the left and then into the right common carotid artery for arterial pressure recordings, followed by LV pressure measurements. All hemodynamic parameters were recorded after a 3-5 min stabilization period using the PowerLab Recorder (ADInstuments), and averaged over 15 consecutive pressure cycles.

For LV *pressure-volume measurements*, mice were anesthetized with urethane (1 g/kg, IP), etomidate (10 mg/kg, IP), morphine (1 mg/kg BW, IP), and pancuronium (1 mg/kg, IP), and mechanically ventilated (MiniVent, Harvard Apparatus). A polyethylene 10 (PE10) catheter was inserted in the left jugular vein for fluid support (bovine serum albumin in physiological saline). The 1.4-F high fidelity pressure-conductance catheter (Millar Instruments) was advanced through the right carotid common artery into the LV. After stabilization of the hemodynamic situation, pressure-volume (PV) loops were recorded (PowerLab Recorder, ADInstruments) while the ventilation was momentarily turned off to avoid respiratory fluctuation of cardiac signals. The parallel volume was determined by bolus injections of 15% sodium chloride solution. The inferior caval vein was multiple times compressed between liver and diaphragm while PV loops were recorded (occlusion loops).

**Protein Extraction and Immunoblot Analysis**

Mouse cardiac tissue was homogenized in Lysing Matrix D tubes (MP Biomedicals) with a Ribolyser (Hybaid), using the modified RIPA lysis solution containing 2 mmol/l Tris-HCl pH 7.4, 137 mmol/l NaCl, 10% glycerol, 1% Triton X-100, 0.1% sodium deoxycholate, 0.1% SDS, 2 mmol/l EDTA pH 7.5, 1 mmol/l Na_3_VO_4_, 2 mM NaF, and Complete protease inhibitor (Roche). The matrix was washed and briefly centrifuged, and the supernatant was transferred to eppendorf tubes, incubated on ice for 30 min, and centrifuged at 13 500 rpm for 10 min. Protein concentrations were determined using a BCA assay (Pierce). Immunoblot analysis was performed with 10-40 μg protein loaded onto a 10% or a 4-12% SDS-PAGE gel, and transferred to nitrocellulose membranes by semi-dry electroblotting. The membranes were blocked for 1 hour in 5% non-fat milk, and incubated overnight with an antibody directed against SERCA2 (Thermo Scientific), PDE5, Erk_1/2_, phosphorylated Erk_1/2_, Akt, phosphorylated Akt (All Cell Signaling Technology), 3-nitrotyrosine (Chemicon International), 4-hydroxy-2-nonenal, and malondialdehyde (both Calbiochem). Bound antibodies were detected using horseradish peroxidase (HRP)-conjugated secondary antibodies developed against the respective primary antibodies (Dako). Peroxidase-labeled secondary antibodies were visualized using ECL Plus western blotting detection reagents (GE Healthcare). Glyceraldehyde-3’-phosphate-dehydrogenase (GAPDH) or actin protein levels were used as a loading control.

**Histological Analysis**

To detect the expression of PDE5 in murine and human myocardial tissue, 6 μm-thick sections were prepared from paraffin-embedded tissue, deparaffinized, and immersed in citrate buffer (Dako) during 20 minutes at 95°C for antigen retrieval. Endogenous peroxidase was quenched by incubation in 0.09% H_2_O_2_ in methanol. Next, sections were incubated with a rabbit anti-PDE5 polyclonal antibody overnight (human tissue: home-made antibody, mouse tissue: Santa Cruz Biotechnology). The bound antibody was detected using an HRP-conjugated secondary antibody (Dako). Signal amplification was obtained using the Vectastain ABC kit (Vector Laboratories), according to the manufacturer's instructions. Exposure of the tissue sections to the chromogene 3,3’-diaminobenzidine tetrahydrochloride/H_2_O_2_ solution, followed by counterstaining with Harris hematoxylin, resulted in visualization of immunoreactivity. Finally, tissue slides were dehydrated, and mounted with glass cover-slides using DPX mounting medium (Prosan).

To confirm PDE5 expression in cardiac myocytes, PDE5 expression was visualized together with desmin (Dako). Alexa Fluor 488 and 568-labeled secondary antibodies were used to detect the primary antibodies (Life Technologies). Images were obtained using an LSM 510 confocal microscope or an Axiovert 200M imaging microscope (Zeiss). After optimization of contrast and brightness settings, pictures were exported without any additional modification.  To determine cardiac myocyte width on laminin-stained tissue sections (Sigma-Aldrich), measurements were obtained at the level of the nucleus in longitudinally sectioned myocytes. To assess the degree of fibrosis in the murine LV, the area of collagen deposition was traced on Sirius red-stained tissue sections using circularly polarized light allowing evaluation of tightly-packed red birefringent collagen and thin, loosely-assembled green birefringent collagen. The degree of fibrosis was expressed as the area of red or green birefringent collagen relative to the area of the examined LV tissue area. The index of apoptosis was determined by dividing the number of immunolabeled cardiac myocyte nuclei by the area of the examined LV tissue area.

**Force Measurements in Isolated Cardiac Myocytes**

Cardiac myocytes were isolated from the LVs of mechanically disrupted frozen hearts. Then, single cardiac myocytes were permeabilized using Triton X-100, and mounted between a force transducer and an electromagnetic motor. Isometric Ca^2+^-contractures were evoked by transferring cardiac myocytes from a Ca^2+^-free relaxing solution (in mmol/l: KCl 37.34, BES 10, MgCl_2_ 6.24, CaEGTA 7, Na_2_ATP 6.99, and sodium creatinin-phosphate 15, pH 7.2) to Ca^2+^-containing activating solutions (of otherwise identical compositions) at a sarcomere length of 2.3 μm, as described previously . Ca^2+^-independent passive force was determined at slack length in relaxing solution. Active and passive force values were standardized to cardiac myocyte cross-sectional area. To determine the Ca^2+^-sensitivity of isometric force production, active forces measured at various [Ca^2+^] were plotted as a function of pCa (pCa=‑log_10_[Ca^2+^]), and a modified Hill’s equation was then employed to determine the [Ca^2+^] evoking half-maximal force production (pCa_50_). To determine cGMP/PKG-dependent modulation of the mechanical function of myofilaments, Ca^2+^-force relationships of cardiac myocytes were determined before and after incubations in relaxing solution supplemented with the catalytic subunit of bovine PKG (PKG-Iα, 0.01 U/mL; Sigma-Aldrich), cGMP (10 μmol/l; Sigma-Aldrich) and dithiothreitol (6 mmol/l; Sigma-Aldrich) for 40 min.

**References**

1. Borbely A, van der Velden J, Papp Z, Bronzwaer JG, Edes I, et al. (2005) Cardiomyocyte stiffness in diastolic heart failure. Circulation 111: 774-781.

2. Papp Z, Szabo A, Barends JP, Stienen GJ (2002) The mechanism of the force enhancement by MgADP under simulated ischaemic conditions in rat cardiac myocytes. J Physiol 543: 177-189.
